# Remote health: what are the problems and what can we do about them? Insights from Australia

**DOI:** 10.1186/s12913-025-12828-0

**Published:** 2025-05-03

**Authors:** Deborah J Russell, John Humphreys, Prabhakar Veginadu, Supriya Mathew, Renee Williams, Sinon Cooney, Leander Menezes, John Boffa, Vahab Baghbanian, April Robinson, Yuejen Zhao, Mark Ramjan, Karrina DeMasi, Walbira Murray, Sean Taylor, Donna-Maree Stephens, Kristal Lawrence, John Wakerman

**Affiliations:** 1 Menzies School of Health Research, Charles Darwin University, Mparntwe (Alice Springs), Northern Territory Australia; 2 School of Rural Health, Monash University, Bendigo, VIC Australia; 3 School of Dentistry and Medical Sciences, Charles Sturt University, Wagga Wagga, NSW Australia; 4 Torres Health Indigenous Corporation, Thursday Island, QLD Australia; 5 Katherine West Health Board, Katherine, NT Australia; 6 Pintupi Homelands Health Service Aboriginal Corporation, Kintore, NT Australia; 7 Central Australian Aboriginal Congress, Mparntwe (Alice Springs), NT Australia; 8 Northern Territory Primary Health Network/Rural Workforce Agency NT, Darwin, NT Australia; 9 Northern Territory Department of Health, Darwin, NT Australia; 10 The Kids Research Institute Australia, Adelaide, SA Australia; 11 Aboriginal Medical Services Alliance Northern Territory, Darwin, Australia; 12 School of Population & Global Health, University of Melbourne, Melbourne, Australia; 13 DMS Research & Evaluation Consulting, Darwin, Australia

**Keywords:** Remote, Rural, Health systems, Primary health care, Equity

## Abstract

This article analyses three broad questions: (i) How is ‘remote’ different from ‘rural’?; (ii) How do these differences affect the provision of health care and health outcomes, positively and negatively?; and (iii) What is needed to address these issues and systematise solutions in order to deliver parity of health outcomes?

## Introduction

The term ‘*rural’* is used, often synonymously with ‘*regional’* in Australia, to denote areas outside of major ‘urban’ and metropolitan centres. As such, the term ‘*rural’* comprises a wide diversity of communities and – ranging from commuter towns and hobby farms proximate to major centres to ‘outback’ settlements, vast rangelands, large pastoral stations, isolated mining towns, First Nations communities, closely settled agricultural settlements and seasonally bustling tourist centres. Historically, considerable work has been done in many countries delineating what constitutes ‘*rural’* from ‘*urban’* [1, 2], including both generic classifications and other taxonomies relating more specifically to some fields of activity such as health [3–5]. In relation to rural health, these designations frequently provide the basis for comparing the health status of residents, access to and outcomes of health care, and the planning and resourcing of health services [6, 7]. Globally, the evidence shows that access to health care and the health outcomes of ‘*rural’* residents are invariably worse than those inhabiting metropolitan centres [8].

However, recent research has demonstrated that in Australia the term ‘*rural’* alone fails to adequately identify or deal with all the health issues characterising vast ‘*remote’* areas of non-metropolitan settlement, also called ‘frontier’ or ‘northern’ in other locations [9–11]. Remote areas have even poorer access to comprehensive health services [12], display far greater inequity in terms of health care resourcing [13], and are also characterised by worse health outcomes, including higher hospitalisation rates [14], than many ‘rural’ areas. Thus, such areas require a significantly more sophisticated health strategy to address these issues. In short, remote health status is arguably even more disadvantaged and problematic than rural health, warranting targeted analysis and action.

To understand this issue, and what to do about it, it is necessary to analyse three broad questions, namely:

i. How is ‘*remote*’ different from ‘*rural’*?;
ii. How do these differences affect the provision of health care and health outcomes, positively and negatively?; and
iii. What is needed to address these issues and systematise solutions in order to deliver parity of health outcomes?

This article addresses these questions by drawing on the extensive body of evidence that has emerged from a nation-wide collaboration of remote health services and researchers based in remote Australia.

### What differentiates ‘remote health’ from ‘rural health’?

In Australia, remote areas are most often differentiated from rural areas using the Accessibility/Remoteness Index of Australia Plus (ARIA+). The ARIA + index scoring for each geographic location is based on road distances to Australian population centres. ARIA + underpins key geographical classifications such as the Australian Statistical Geography Standard (ASGS) which comprises five categories, two of which represent ‘*remote*’ i.e. category 4 ‘Remote’ and category 5 ‘Very Remote’. The distinctions between ‘*remote*’ and ‘*rural*’ health are crucial, though often inadequately considered in health policy. By definition, geographical isolation is greater in remote than rural areas [15, 16]. Climatic conditions are also frequently more extreme [17]. Invariably settlements are diverse, dispersed, smaller, and lack economies of scale for services [18]. These features mean that different workforce and service delivery models are required. Types of economic activities and how they are structured differ in remote locations, with visiting, outreach, or increasingly virtual services supplementing limited local in-person services [19]. Remote areas have a higher proportion of socio-economically and educationally disadvantaged communities with populations frequently experiencing marked poverty and overcrowding in substandard housing infrastructure [20]. Remote areas also have a higher proportion of First Nations people with strong connections to country, culture and kin, amidst a backdrop of troubled histories of colonisation, disempowerment and intergenerational trauma [16, 21]. The lack of population critical mass and minority status of remote populations mostly limits political clout.

Remote areas also often experience greater difficulties with workforce supply and retention [22], and subsequently workforce composition tends to differ from rural (e.g. Remote Area Nurses (RANs), Aboriginal Health Practitioners (AHPs) and other cadres substituting for General Practitioners (GPs)), as does skill requirements (e.g. greater need for public health, comprehensive primary health care (PHC) and emergency skills). Remote health providers typically experience greater professional and social isolation than rural health providers [16]. Poorer access to services results from geographical distance, climatic factors such as seasonally flooded and impassable roads, poorer transportation infrastructure, economic disadvantage and availability of an appropriately-skilled workforce [16]. Nevertheless, borne of scarce resources relative to need, remote is characterised by a high level of innovation in providing services to remote settlements.

### How do these differences affect health outcomes and the provision of health care?

The characteristics that differentiate ‘remote’ communities from ‘rural’ centres are noteworthy contributors to several significant differences in the health status of their inhabitants and the nature and adequacy of the health care services available to meet their needs.

i. Compared with metropolitan, regional and rural centres, Australians living in remote and very remote areas [3] exhibit different and *greater morbidity*,* higher avoidable mortality*,* and lower uptake of preventive care programs*. Life expectancy at birth, for example, is 10.6 years shorter for persons living in remote Northern Territory (NT) compared to those living in Greater Sydney; [14] potentially avoidable deaths are 1.8 and up to 2.8 times higher for remote and very remote populations, respectively, compared to major cities [14]. Remote and very remote populations have much greater rates of death due to chronic diseases such as coronary heart disease (1.4 and 1.7 times), diabetes (1.7 and 3.5 times) and rates of suicide (1.5 and 2.0 times) than the Australian average [14]. In particular, for First Nations populations living in remote and very remote Australia, the health outcome disparities are immense [23, 24]. For example, diabetes prevalence amongst remote Central Australian First Nations adults (age > 20) at 40% is amongst the highest in the world, yet treatment is suboptimal for the majority [25].
ii. These health outcome inequities are not surprising given that residents in remote and very remote areas of Australia have markedly *greater socioeconomic disadvantage* with up to 30% of health inequities in the NT First Nations population attributable to socioeconomic disadvantage [23].
iii. In remote areas, higher population health needs are met with *poorer access to PHC services* compared with regional areas or major cities [26]. Poorer access to, and decreased utilisation of PHC services is associated with poorer health outcomes [27, 28]. Geographic disadvantage not only affects access to and cost of delivering services, but also the efficient utilisation of resources and *equity of funding*. Where access to PHC is low, for example due to lack of healthcare professionals such as AHPs, GPs and RANs, or failures to provide culturally safe care, patients have reduced access to preventive services, resulting in delay in treatment and increased emergency presentations, evacuations and preventable hospitalisations. The additional cost associated with delivering health services in remote areas and the extra time and resources needed to ensure culturally safe care, may mean that revenue is insufficient to sustain remote health service delivery. Current PHC funding models such as reliance on Fee-For-Service billing via Australia’s Medicare Billing Scheme and the National Disability Insurance Scheme (where income is directly dependent on practitioner availability and preparedness to provide frequent, brief services) are manifestly inadequate and further exacerbate inequities.
iv. Moreover, *climate change* adversely affects important health risk factors including water quality, food security, energy poverty, adequacy of housing and telecommunications in remote Australia. This increases the need for appropriate (and usually more expensive) climate-resilient health systems [29].

### What is needed to ensure parity of health outcomes regardless of remoteness?

We know what to do – there is evidence about what works, where it works and why. This is not a matter of lack of knowledge. It is a failure to translate our current knowledge into policy and reflects unwillingness amidst short-term political cycles to make the necessary investments needed for longer term improvements amidst prevailing racist, metro-centric and self-interest agendas of the majority. This is despite a high capacity for these populations to benefit.

Despite the constraints and challenges presented by *‘remote’* areas in relation to providing accessible and equitable health care, these areas have often been significant incubators of ‘innovative’ solutions in a tough, resource-poor context. Innovations have included imaginative models of PHC such as combining Fly-In/Fly-Out visiting services and telehealth [29], developing more equitable funding models [30], workforce supply and employment and training arrangements [31], and preventive health programs [32]. However, despite significant innovation and accompanying evidence of effectiveness, there has been failure by government to fully ‘take-up’ the evidence provided by various ‘pilots’ and ‘trials’ in any comprehensive systemic remote health strategy. In the absence of an appropriate strategy to guide the provision of appropriate, accessible and affordable comprehensive primary health services, residents in remote areas of Australia (particularly First Nations peoples) will increasingly experience high rates of preventable disease and premature mortality. Evidence shows that overcoming many of the existing barriers to service access is neither insurmountable nor excessively expensive. For example, the savings from reducing the currently excessive workforce turnover more than cover the cost of recruiting agency staff, training and incentives [33].

To ensure sustainability, a genuine remote health strategy based on full community engagement and government commitment is needed that addresses systemic issues rather than an ad hoc approach, while at the same time recognising the diversity of needs and contexts that characterise ‘*remote’* communities. This strategy should incorporate and outline all those components that are needed anywhere to ensure appropriate sustainable PHC service [19, 34]. Central among the integrated components underpinning such a remote health strategy are:

i. *Funding*: Given that resources are key to the provision of adequate, appropriate, and accessible PHC services, a different funding model that is based on health needs rather than practitioner availability, and thus takes account of the context of ‘remote’, is required to ensure genuine equity in resource allocation and distribution;
ii. *Workforce education*,* training and supports*: Local (including On Country), contextualised education is needed, with ongoing training and supports. These enable remote health care workers to provide high quality, culturally safe health care. Local ownership and community consultations that reflect the education and training continuum are also required to maximise local recruitment and retention;
iii. *Different workforce scope-of-practice* to ensure whole-of-patient care;
iv. *Recognised First Nations leadership roles* in remote PHC;
v. *Context-specific service models* that take account of local health needs, cultural differences, and lack of economies of scale;
vi. *Genuine inter-sectoral collaboration and resourcing* to link health care with housing, employment, education, justice, transport among others;
vii. * Appropriate climate-resilient remote infrastructure and reliable Information Technology and telecommunications; and*.
viii. *Strong engagement and partnerships with local communities*.

Table 1 provides examples of each of these components.

**Table 1:** Examples of integrated components underpinning a remote health strategy

Integrated components underpinning a remote health strategy	Example
Funding	Primary Health Care Access Program (PHCAP) which was a flexible mixed mode pooled funding model implemented via a grant payment plus access to Medicare Benefits Schedule and Pharmaceutical Benefits Scheme payments [30]
Workforce education, training and supports	Mental health and social supports such as the 24/7 telephone counselling support line available through the Bush Support Line; [35] and career development opportunities provided, including in management.
Different workforce scope-of-practice	Services for Australian Rural & Remote Allied Health’s (SARRAH’s) ‘Building the rural and remote Allied Health Assistant workforce’ (BRAHAW) initiative which enables remote health organisations to develop their Allied Health Assistant workforce [36]
Recognised First Nations leadership roles	The Torres Model of Care has a First Nations leadership model and approach which enables trust and support from both the community and the health sector [37]
Context-specific service models	Acknowledging and integrating traditional healing practices alongside Western medicine [38]
Genuine inter-sectoral collaboration and resourcing	Collaborations occurring between health sector and essential services including police and education departments during the COVID-19 pandemic [39]
Appropriate climate-resilient remote infrastructure and reliable Information Technology and telecommunications	Culturally sensitive and climate-friendly housing in remote communities [40]
Strong engagement and partnerships with local communities	Aboriginal and Torres Strait Islander Community Controlled Health Services governance models which are controlled and delivered by the people they serve [41]

The remote health strategy should identify an implementation plan outlining who is responsible, how much will it cost, timelines identifying pre-requisites, and political and economic risk assessment. In addition, it should be accompanied by an evaluation strategy with performance indicators to monitor what is working well and those factors inhibiting progress towards achieving targeted health outcomes.

## Conclusion

The importance and implications of how *‘rural’* and *‘remote’* areas are delimited and differentiated for the assessment of health needs and resource allocation cannot be overestimated. The distinguishing characteristics of remote areas warrant a strategic approach to health care that takes account of their impact on health status and the delivery of services. Such action will only occur if there is more advocacy and agitation at the highest political levels of government, and better knowledge translation so that bureaucrats and politicians can ‘take up’ appropriately contextualised research evidence more readily in the policy arena. The health and wellbeing benefits to the population and the cost savings associated with reducing evacuations and avoidable hospitalisations could far outweigh the harms of persisting with the existing largely reactive and ad hoc approach to addressing the health needs of remote area residents, though further research is needed to confirm this. Given increasing societal demands for health care and finite public resources, without such a remote health strategy things will only get worse and health status inequalities increase.

## Data Availability

No datasets were generated or analysed during the current study.

## Abbreviations

GP: General practitioner
NT: Northern Territory
PHC: Primary health care
RAN: Remote area nurse
AHP: Aboriginal health practitioner
